# Fish bone migration into urachus mimicking urachal malignancy: A rare case report

**DOI:** 10.1016/j.radcr.2025.10.045

**Published:** 2025-11-17

**Authors:** Sajad Ahmad Para, Tufeel Ahmad Khan, Abdul Rouf Khawaja, Naseer Ahmad Choh, K. Gokul, Syed Shakeeb Arsalan

**Affiliations:** a Department of Urology, Sher-i-Kashmir Institute of Medical Sciences, Srinagar India; b Department Radiodiagnosis and imaging, Sher-i-Kashmir Institute of Medical Sciences, Srinagar, India

**Keywords:** Bladder neoplasms/diagnostic imaging, Fish bone, Foreign bodies/migration, Urachal carcinoma

## Abstract

Foreign bodies can find their way into and around the bladder usually via urethra by self insertion, migration from surrounding structures and by penetrating trauma. The clinical presentation being varied, symptoms nonspecific and history often concealed makes diagnosis quite difficult. Fish bone ingestion has been reported to caused bowl perforation and rarely migrate to other surrounding structures. We present a case of 39 year old male who presented to us with LUTS and lower abdominal mass. Evaluation revealed a mass extending from the dome of urinary bladder to umbilicus. Imaging modalities could not certainly characterize the nature of this lesion. Contrast CT revealed a large, irregular, enhancing mass with thin streak of central calcification and necrosis raising the possibility of urachal malignancy. The mass was excised enblock along with dome of urinary bladder and revealed a long fish bone in the center of specimen. Foreign bodies in the urachus are very rare and may present as inflammatory masses which are difficult to differentiate from the malignant pathology. Imaging when augmented with proper history and clinical examination plays a crucial role in the identification of these foreign bodies and helps to differentiate these inflamatory masses from more sinister malignant pathologies.

## Introduction

Foreign bodies in and around the bladder always pose a clinical challenge to accurately diagnose and treat such rare ailments. Most of these objects are self administered via urethra while as others migrate from surrounding organs or find place in bladder after penetrating trauma. Self insertion is more common in men and is usually associated with psychiatric illness or to obtain sexual gratification. Foreign bodies have been found to migrate into urinary bladder from surrounding structures and include orthopedic screws, intrauterine contraceptive devices, fish and chicken bones [1]. Patent urachus remains a rare place for any foreign body to settle in and usually enters via umbilicus. Intact fish bones are ingested accidentally and most of them pass out over a period of 5-7 day without any complications. Being sharp and thin, these bones can cause perforation of bowel leading to frank peritonitis or a localized intraabdominal abscess formation. This complication is rare occurring in about 1% of patients and usually involves duodenojejunal, ileocecal or rectosigmoid junction because of associated sharp turns facilitating extraluminal migration [2]. Upon perforating bowel these bone usually stay extraluminal and rarely migrate into other surrounding hallow viscus like urinary bladder. We report a very rare case of migration of fish bone into patent urachus give rise to a large inflammatory mass mimicking urachal malignancy.

## Case report

**Fig. 1:**
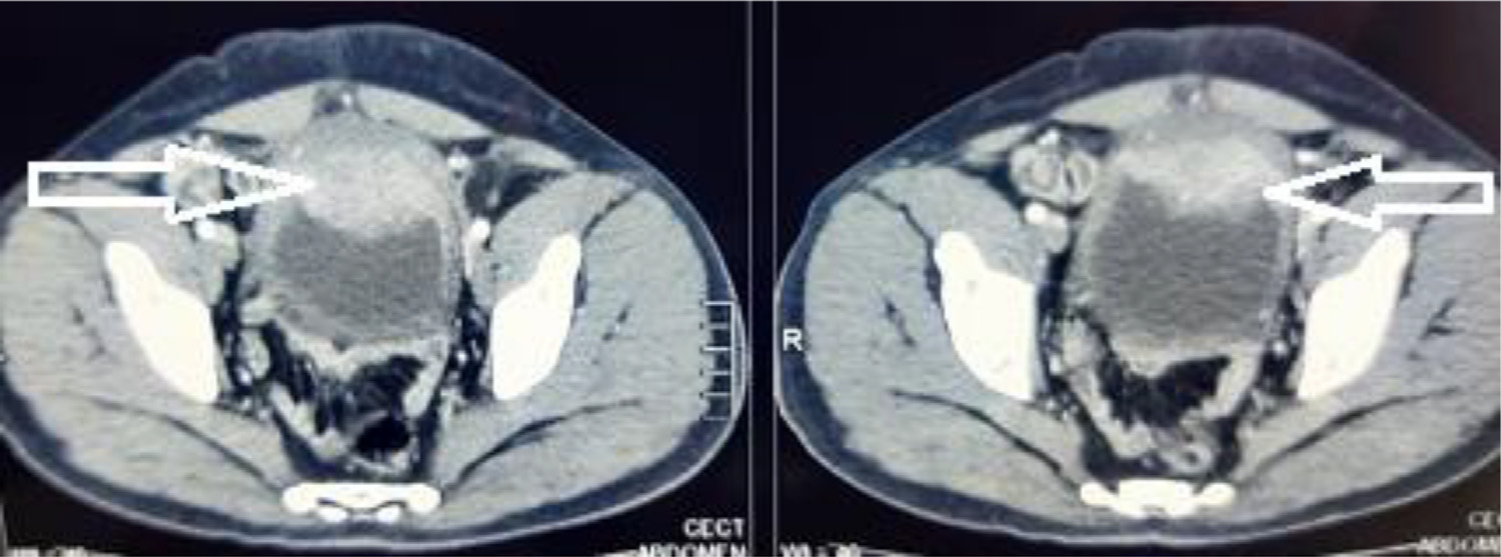
Axial contrast CT showing an irregular 7 × 8 cm enhancing mass related to dome of bladder (white arrow) with surrounding fat stranding.

**Fig. 2:**
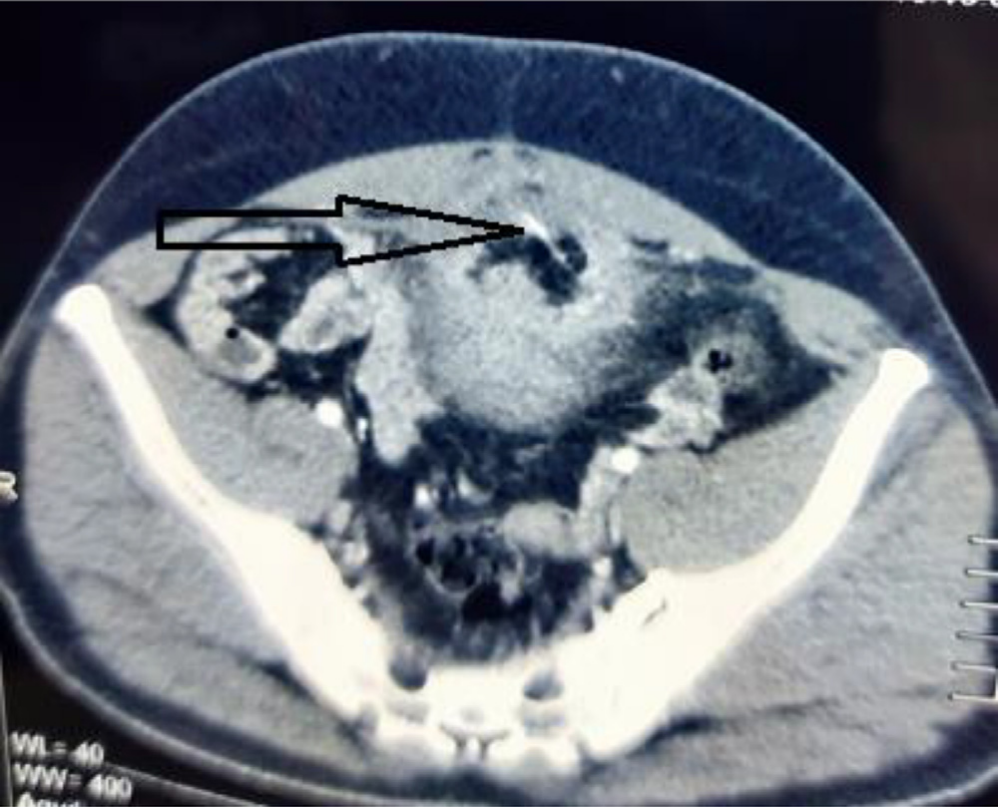
Axial CT image showing mass with linear calcified streak and central necrosis (white arrow).

**Fig. 3:**
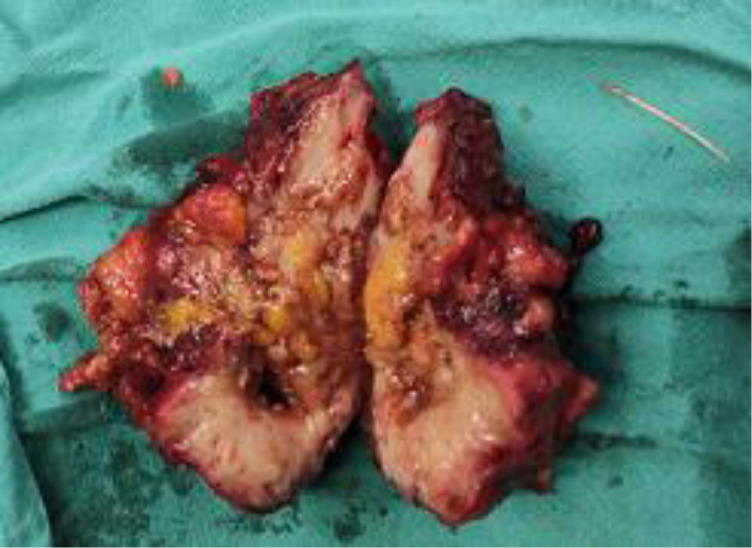
Specimen of mass excised along with dome of the bladder.

**Fig. 4:**
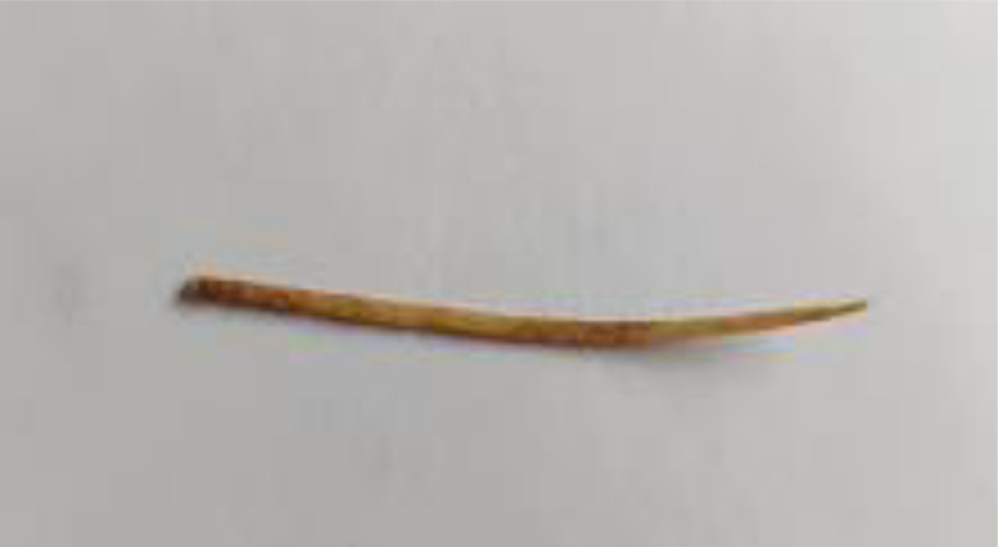
Fish bone retrieved from specimen.

We report the case of 39 years old male who presented to us with complaints of lower abdominal pain, increased urinary frequency, dysuria and progressively increasing infraumbilical swelling for 1 month. There was no hematuria or any other significant history. On palpation, a hard lump with mild tenderness was palpable just below umbilicus and the overlying skin was absolutely normal. Initial ultrasound (USG) examination revealed a solid mass lesion, 7 × 8 cms in relation to dome of bladder extending to umbilicus. There was central area of liquefaction raising the possibility of inflammatory mass with abscess formation or urachal malignancy with central necrosis. Cross section computed tomography (CT) imaging of the patient revealed an irregular, enhancing, solid mass, 6 × 7 cms involving dome of urinary bladder with surrounding fat stranding and abutting the anterior abdominal wall (Fig. 1). There was a thin linear calcified streak, 2.5 cms in length in the middle of the lesion with surrounding necrosis (Fig. 2). Cystoscopic examination of the patient showed a broad based solid growth on the dome of bladder, raising the possibility of urachal cancer. Patient was planned for laparoscopic partial cystectomy with excision of urachal mass. On laparoscopy, it was noted that there was a mass lesion involving the dome of bladder and extending to umbilicus. The small bowl and sigmoid colon were adherent to it and there was no plane between mass and anterior abdominal wall. The small bowl adhesions were released but sigmoid colon could not be dissected away from mass because of dense adherence. Decision to convert to open surgery was made and abdomen was opened by a midline incision. Mass was excised along with dome of bladder (Fig. 3). On opening the specimen a sharp fish bone (2.5 cms) was retrieved (Fig. 4). Bladder was repaired primarily and procedure was uneventful. When history was reviewed, patient revealed that he had taken fish two months back and did not notice accidental fish bone ingestion. After that patient remained absolutely asymptomatic till he developed lower urinary tract symptoms after 2 months. The dense adherence of sigmoid colon to the mass raised the possibility of migration of fishbone from sigmoid colon to urachus. Histopathology of the specimen revealed inflammatory pathology with foreign body granuloma without any evidence of malignancy.

## Discussion

In adults, accidental ingestion of foreign bodies is usually food related and fish bone is one of the commonly swallowed sharp objects. Although thin and sharp, most of the ingested bones pass out uneventfully over the period of 5-7 days. However gut perforations have been reported in less than 1% of cases. The most common site of perforation is terminal ileum followed by rectosigmoid junction, attributed to sharp anatomical turns [3]. These perforations may present as acute abdomen with signs of peritonitis or slowly developing intraabdominal collection presenting with fever and long standing abdominal pain. In rare instances fish bones had been reported to cause bladder perforation, duodenocaval fistula, enterocutaneous fistula, hepatic abscess and hepato-enteric fistula [4]. In our case patient had ingested fish 2 months back and did not notice swallowing of intact fish bone. Following that patient was absolutely asymptomatic till recently when he developed lower urinary tract symptoms (LUTS) and infraumbilical swelling. It had most likely migrated from rectosigmoid junction into patent urachus since there were dense adhesions between these two structures. Kodoma K et al. reported endoscopic drainage of bladder wall abscess caused by fish bone stuck within bladder wall following migration from small bowel [5]. Alanazi AB et al. has reported a similar case of fish bone in patent urachus mimicking as urachal malignancy [6]. In their case patient resented with fever, LUTS and lower abdominal swelling.

X –Ray radiography has been commonly employed to localize ingested foreign bodies. Utility of plain radiography is limited due to its inability to detect radiolucent objects and less sensitivity to identify small caliber foreign bodies like fish bones. Inflammatory urachal masses are difficult to differentiate from malignant pathologies on ultrasound in absence of clinical signs and symptoms. None the less it is the first investigation which is used to assess the urachal swellings. On grey scale ultrasonography, the inflammatory pathologies appear as hypoechoic masses in the extraperitoneal space extending from bladder dome to umbilicus with internal echoes representing abscess formation. Urachal malignancies appear as hyperechogenic soft tissue lesions with internal vascularity demonstrated on color doppler imaging [7]. Computed tomography with contrast is the gold standard imaging modality to diagnose and localize the foreign bodies. It provides the detailed imaging of organ involved, three dimensional relationship of foreign body to surrounding structures and any associated complications [8]. Besides that it readily differentiates a malignant pathology from the inflammatory ones in most of the cases. On CT, inflammatory urachal pathologies appear as irregular masses with peripheral enhancement and central nonenhancing low attenuation. Neoplastic lesions appear as well defined solid-cystic masses located between dome of bladder and umbilicus with irregular enhancing wall. Malignant urachal lesions usually demonstrate low attenuation in 60% of cases due to presence of necrosis or mucin [9]. In our case CT revealed a large enhancing mass with thin linear calcification involving dome of bladder and urachus. Close proximity of the calcified streak to the umbilicus created skepticism about self insertion of foreign body but same was denied by patient. Clinical signs and radiological features were more in favor of urachal carcinoma than any other pathology. On magnetic resonance imaging (MRI), infected urachal swellings appear as irregular supravesical masses with variable signal intensity and nonhomogeneous contrast enhancement. Malignant urachal lesions may be predominantly cystic in nature demonstrating high T_2_ signal or multilobulated with cystic and solid components in different proportion demonstrating intermediate and high T_2_ signal depicting necrosis, cystic changes or presence of mucin. Urachal cancers demonstrate nonhomogenous enhancement on post contrast T_1_ weighted images [10]. Despite all advancements in imaging, malignant urachal pathologies cannot be surely differentiated from inflammatory lesions. Combination of a proper history taking, clinical examination and appropriate cross section imaging help to reach the diagnosis and plan management.

The incidence is urachal cancer has been reported between 0.01%-0.02% in literature and accounts for less than 1% of bladder cancers. Surgical management consists of enbloc resection of urachus along with bladder dome [11]. In our case, whole mass was resected along with bladder dome. Upon opening the specimen, fish bone was retrieved. Histopathology revealed inflammatory pathology with foreign body granuloma without any evidence of malignancy. Patient had uneventful recovery, requiring anticholinergic medication initially for urinary frequency which settled over a period of 6 weeks.

## Conclusion

Accidentally ingested fish bones usually traverse the gut uneventfully, but at times can lead to life threatening complications. Migration of fishbone from gut into intact urachus is extremely rare and imaging plays a crucial role in identification of these objects and accessing collateral damage. Besides that it becomes clinically imperative to differentiate these inflammatory masses from malignant pathologies to plan appropriate management. Contrast enhanced CT plays a key role in identifying these foreign bodies, assessing organ injury and differentiating these inflammatory masses from malignant ones.

## Author contributions

Sajad Ahmad Para- Data Collection and follow up of patient, Tufeel Ahmad Khan –photographs, Abdul Rouf Khawaja-review of literature, Naseer Ahmad Choh-Design, Gokul K- supervision, Syed Shakeeb Arsalan- follow up of patient.

## Patient consent

Written informed consent was taken from the above mentioned patient for publishing the data. The work is approved by the institution ethical committee with protocol number of IEC/SKIMS EC321/2025.

## References

[1] Shimokihar K., Kawahara T., Hayashi Y., Tsutsumi S., Takamoto D., Mochizuki T. (2017). Foreign body in the bladder: a case report. Int J Surg.

[2] Eckford S.D., Persad R.A., Brewster S.F., Gingell J.C. (1992). Intravesical foreign bodies; five year review. Br J Urol.

[3] Singh R.P., Gardner J.A. (1981). Perforation of the sigmoid colon by swallowed chicken bone: case reports and review of literature. Int Surg.

[4] Chen H.K., Kuo J.R., Uen Y.H. (2011). Liver abscess secondary to fish bone migration from the duodenum. ANZ J Surg.

[5] Kodama K., Ofude M., Motoi I., Hinoue Y., Saito K. (2010). Successful endoscopic management of fish bone embedded into the bladder wall. Case Rep Med.

[6] Alanazi A.B., Aldhowayan A.M., Almuhanna M.M. (2022). Fish bone perforation into a patent urachus mimicking urachal carcinoma: case report. Urol case rep.

[7] Luo X., Lin J., Lianfang Du, Rong Wu, Zhaojun Li (2019). Ultrasound findings of urachal anomalies. A series of interesting cases. Med Ultrason.

[8] Ninitas P., Anselmo M., Silva A., Ferreira A., Santos J. (2019). Urachal abscess mimicking malignant tumor: can imaging tell them apart?. Acta Radiologica Open.

[9] Das J.P., Vargas H.A., Lee A., Hutchinson B., O'Connor E. (2020). The urachus revisited: multimodal imaging of benign & malignant urachal pathology. Br J Radiol.

[10] Khati N.J., Enquist E.G. (1998). Javitt MC imaging of the umbilicus and periumbilical region. Radiographics.

[11] Hayashi T., Yuasa T., Uehara S. (2016). Clinical outcome of urachal cancer in Japanese patients. Int J Clin Oncol.

